# Crosstalk Between ROS and Autophagy in Tumorigenesis: Understanding the Multifaceted Paradox

**DOI:** 10.3389/fonc.2022.852424

**Published:** 2022-03-10

**Authors:** Adria Hasan, Suroor Fatima Rizvi, Sana Parveen, Neelam Pathak, Aamir Nazir, Snober S. Mir

**Affiliations:** ^1^ Molecular Cell Biology Laboratory, Integral Information and Research Centre-4 (IIRC-4), Integral University, Lucknow, India; ^2^ Department of Bioengineering, Faculty of Engineering, Integral University, Lucknow, India; ^3^ Department of Biosciences, Faculty of Science, Integral University, Lucknow, India; ^4^ Department of Biochemistry, Dr. RML Avadh University, Faizabad, India; ^5^ Laboratory of Functional Genomics and Molecular Toxicology, Division of Neuroscience and Ageing Biology, CSIR-Central Drug Research Institute, Lucknow, India

**Keywords:** autophagy, ROS, tumor microenvironment, epithelial–mesenchymal transition, metastasis, anticancer therapy resistance

## Abstract

Cancer formation is a highly regulated and complex process, largely dependent on its microenvironment. This complexity highlights the need for developing novel target-based therapies depending on cancer phenotype and genotype. Autophagy, a catabolic process, removes damaged and defective cellular materials through lysosomes. It is activated in response to stress conditions such as nutrient deprivation, hypoxia, and oxidative stress. Oxidative stress is induced by excess reactive oxygen species (ROS) that are multifaceted molecules that drive several pathophysiological conditions, including cancer. Moreover, autophagy also plays a dual role, initially inhibiting tumor formation but promoting tumor progression during advanced stages. Mounting evidence has suggested an intricate crosstalk between autophagy and ROS where they can either suppress cancer formation or promote disease etiology. This review highlights the regulatory roles of autophagy and ROS from tumor induction to metastasis. We also discuss the therapeutic strategies that have been devised so far to combat cancer. Based on the review, we finally present some gap areas that could be targeted and may provide a basis for cancer suppression.

## Introduction

Autophagy, meaning “self-eating,” is a catabolic process where cytoplasmic organelles, proteins, and other macromolecules are degraded during starvation or other types of stress (1–3). It is vital in maintaining cellular homeostasis, helps eliminate pathogens, and is regulated by the autophagy-related (ATG) genes. The molecules/cargo to be degraded are sequestered in double-membrane vesicles (autophagosomes). Autophagosomes fuse to lysosomes, forming autolysosomes that lead to cargo degradation. The degraded molecules provide energy that can be used in anabolic and bioenergetic pathways (4). Apart from macroautophagy, there are two other forms of autophagy: microautophagy and chaperone-mediated autophagy (5). Any disruption in autophagic pathways has been shown to play a significant role in different diseases such as neurodegeneration, atherosclerosis, and cancer (6, 7).

Usually, autophagy acts as a tumor suppressor during initiation but promotes cancer cell proliferation in established tumors (8). Autophagy can be regulated by several factors, including starvation, infections, drugs, hypoxia, ATP/AMP ratio, and reactive oxygen species (ROS) levels (9). Cancer cells also exhibit high ROS levels (10) due to increased metabolism rate, incomplete oxidative phosphorylation, mitochondrial dysfunction, low nutrient levels, hypoxia, and low pH in their microenvironment (11–13). Under normal conditions, low ROS levels are generated to regulate signaling pathways, including autophagy, to maintain cellular homeostasis (14–16). Moreover, starvation conditions known to upregulate autophagy can also induce ROS. Consistently, studies have shown ROS-mediated regulation of autophagy as ROS scavengers or high expression of antioxidants can block stress-induced autophagy (17, 18).

ROS-induced autophagy can lead to cell death or survival (17, 19). High ROS levels can also activate several oncogenic pathways, such as mitogen-activated protein kinase (MAPK) and nuclear factor (NF)-κB signaling pathways. Contrarily, increased ROS can also promote cell death by activating the tumor suppressor p53 or apoptosis caused by excessive mitochondrial and DNA damage (20). Thus, an intricate cellular balance between autophagy and ROS is required to maintain cellular redox balance in normal and disease-related physiological conditions. Therefore, the exact role of autophagy and ROS in cancer cells is context-dependent and varies in different cancer phenotypes (21–24). This review describes the role of autophagy and ROS as tumor promoters and suppressors. We further discuss the intricate crosstalk between autophagy and ROS that can regulate tumor promotion, metastasis, and response to therapy and may ultimately decide the fate of cancer cells.

## Regulation of Autophagy

**Figure 1:**
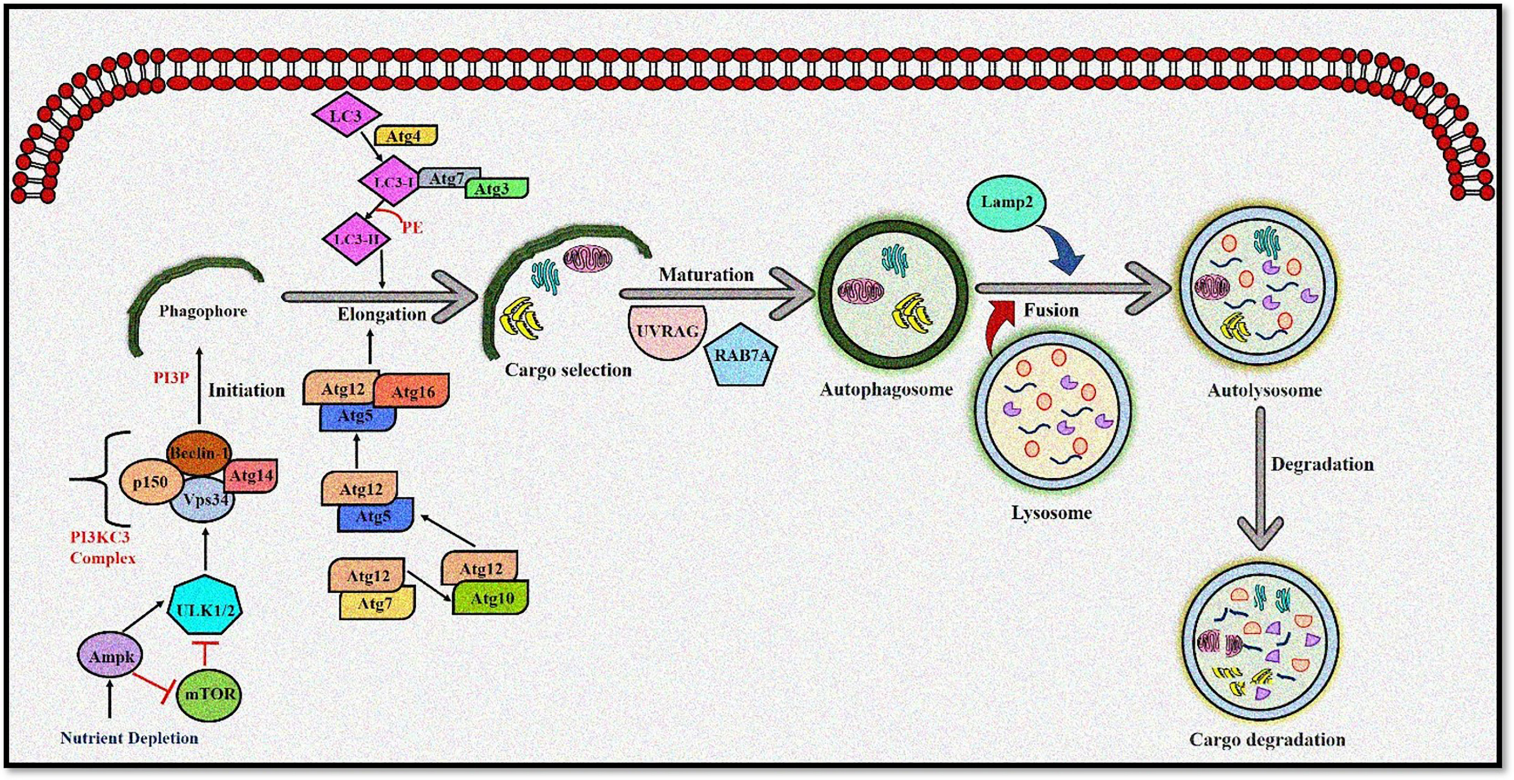
The autophagy pathway. AMPK and ULK1 kinase complex initiates autophagy. mTOR inhibition promotes phagophore formation through class III PI3K and Beclin 1 complex formation. Atg5-12 complex and LC3 are required to complete the autophagosome. After maturation, autophagosomes fuse with lysosomes to form autolysosomes where cargo degradation occurs. UVRAG, RAB7A, and LAMP2 mediate autophagosome maturation and fusion with lysosomes. AMPK, 5' adenosine monophosphate-activated protein kinase; ULK1, Unc-51 Like Autophagy Activating Kinase 1; mTOR, mammalian target of rapamycin; PI3K, phosphoinositide 3-kinase; ATG, Autophagy related; LC3, Microtubule-associated protein 1A/1B-light chain 3; UVRAG, UV radiation resistance-associated gene protein; RAB7A, Ras-related protein Rab-7a; LAMP2, lysosomal-associated membrane protein 2.

Autophagy is moderately active at the basal level but becomes highly activated due to different cellular stresses, including chemotherapeutics and radiotherapy (25–27). To date, 35 different *ATG* genes have been identified in yeast that are also conserved in higher eukaryotes (28–31). The autophagy pathway can be divided into several steps: (a) initiation and nucleation, (b) autophagosome closure, (c) maturation through autophagosome–lysosome fusion, and (d) cargo degradation through lysosomal enzymes. Autophagy is regulated through a series of proteins, including mammalian target of rapamycin (mTOR) and 5' adenosine monophosphate-activated protein kinase (AMPK). Activated mTOR negatively regulates autophagy through phosphorylation of the Atg proteins. However, during stress conditions, mTOR is inhibited, and autophagy is enhanced. Conversely, AMPK negatively regulates mTOR and induces the autophagic process (32; 33). After mTOR inhibition, the Unc-51-like autophagy-activating kinase (ULK) complex is activated (34), which in turn activates the class III phosphoinositide 3 kinase (PI3K) (35). The class III PI3K complex consists of several proteins including VPS34, p150, Atg14, and Beclin-1, which initiates autophagosome formation. Beclin-1, a primary autophagy regulator, recruits different proteins involved in the maturation and elongation of the autophagosome. Subsequently, Atg9 protein mediates the trafficking of the source membrane for autophagosome elongation. These may include the Golgi complex, mitochondria, endoplasmic reticulum, endosome, and plasma membrane (36). The primary component required for autophagosome maturation is the ubiquitin-like protein lipidation system that conjugates phosphatidylethanolamine to the C terminus of Atg8 (LC-3) protein, thereby facilitating the incorporation of Atg8 protein into autophagosomal membranes (37, 38). The proteins Atg7 and Atg10 help in conjugating Atg12 protein to Atg5 protein. The Atg12–Atg5 protein complex then conjugates with Atg16L1 protein to promote Atg8 protein lipidation. Atg8 protein is present in the inactive pro-Atg8 form but is cleaved by Atg4B protein, leaving a C-terminal glycine residue (39). The lipidated form of Atg8 protein is strongly associated with the autophagosomal membranes. Yeast contain a single Atg8 protein, while mammals have seven Atg8 proteins in two structurally related subfamilies (MAP1LC3A, B, C and GABARAP, GABARAPL1, and GABARAPL2), signifying a complex diversification of their functions (37). During autophagy induction, damaged organelles, protein aggregates, and ubiquitinated proteins are sorted to the phagophore for degradation. The Atg5–Atg12–Atg16L protein complex localizes to the phagophore, forming a cup-shaped structure, and dissociates when LC3-II localizes to the phagophore to complete the autophagosome formation. The cargo adaptor proteins like p62, NBR1, or NIX are further recruited on the autophagosome to target ubiquitinated protein aggregates and damaged organelles for degradation (40–42). Furthermore, the autophagosome fuses with the lysosomes forming autolysosomes to degrade targeted contents (**Figure 1**). This fusion is mediated by lysosomal-associated membrane protein 2 (LAMP2), the small GTPase RAB7A and UVRAG. Finally, lysosomal hydrolases and cathepsins degrade the targeted proteins, while cathepsins degrade LC3-II on the inner autophagosomal surface (43).

## Reactive Oxygen Species and Autophagy

ROS include a reactive group of molecules such as hydroxyl radical, superoxide anion $\left(O_{2}^{-}\right)$ , and hydrogen peroxide (H_2_O_2_) (44). During normal physiological conditions, most intracellular ROS are produced in the mitochondria during oxidative phosphorylation due to the leaking of electrons from the electron transport chain (45, 46). However, an increase in intracellular ROS levels can promote mitochondrial dysfunction by accumulating high ROS levels, oxidation of lipids, proteins, and DNA damage (**Table 1**) (56, 57). The selective removal of damaged mitochondria by autophagy is called mitophagy. It is mediated by two signaling pathways, namely, NIX/BNIP3L and PARKIN (PARK2)/phosphatase and tensin homolog (PTEN)-induced putative kinase 1 (PINK1) (58–61). Targeting mitochondria toward mitophagy requires interaction between Nix/BNIP3L and GABARAP at the autophagosome (41, 62). PARKIN/PINK1 help remove dysfunctional mitochondria in response to ROS-induced mitochondrial membrane depolarization (63). Furthermore, the redox balance in a cell is maintained through the antioxidant defense system consisting of glutathione peroxide (GPx), catalase, glutathione reductase, glutathione S-transferase (GST), superoxide dismutase (SOD), and glutathione (64). Intracellular H_2_O_2_ is generated by SOD-catalyzed dismutation from $O_{2}^{-}$ formed within the mitochondria (46). Increased H_2_O_2_ levels were observed during tumorigenesis due to increased ROS production, high SOD levels, and inactivation of H_2_O_2_-scavenging enzymes (48). High H_2_O_2_ levels induce autophagic cell death in glioma cells after treatment with the polycyclic ammonium ion sanguinarine, which increases electron leakage from mitochondria and induces NADPH oxidases (NOXs) (65). NOXs, a membrane-bound enzyme complex, is another source of extracellular ROS (49) and are abnormally upregulated in cancer cells (66).

**Table 1:** Role of different reactive oxygen species in cancer.

	ROS	Roles in Cancer	References
1	Generic ROS	Activation of oncogenes. Activate oncogenic signals including Ras, Bcr-Abl, c-Myc, which hyperactivates cell proliferation. Inactivation of tumor suppressors, promoting angiogenesis, and mitochondrial dysfunction. Induction of Wnt/β-catenin pathway which increases metastatic potential. High expression of MMPs. Matrix metalloproteinases (MMPs) trigger epithelial-mesenchymal transition (EMT) MMPs inhibitor or ROS inhibitor may be useful in the reversal of EMT or the killing of cancer stem cells. Regulation of NF-κB pathways Contribution to drug resistance such as through high mutagenic rates	(47–49)
2	Hydrogen Peroxide (H_2_O_2_)	Promotes phosphoinositide 3 kinases (PI3Ks)/RAC-alpha serine/threonine-protein kinase (Akt) survival pathway. Enhanced MAPK and ERK signaling pathway. Oxidative modification of PTEN Oncogenic stabilization of hypoxia-inducible factor (HIF)-1α; conversion to hydroxyl radical	(50; 51, 52)
3	Hydroxyl radical (•OH)	Initiates lipid peroxidation promotes DNA mutagenesis	(53, 54)
4	Hypochlorous acid (HOCl)	Induces mutations in mitochondrial DNA with age	(54)
5	Superoxide anion $\left(O_{2}^{-}\right)$	Conversion to H_2_O_2_, peroxynitrite Stimulates AMPK activity to induce metastasis. Oncogenic stabilization of HIF-1α	(51, 55)

Ras, Rat sarcoma virus; Bcr-Abl, breakpoint cluster region protein -v-abl Abelson murine leukemia viral oncogene; c-Myc, Cellular myelocytomatosis oncogene; MAPK, Mitogen-activated protein kinase; ERK, extracellular-signal-regulated kinase; PTEN, Phosphatase and Tensin Homolog deleted on Chromosome 10; DNA, deoxyribonucleic acid; H2O2, Hydrogen peroxide; AMPK, 5' adenosine monophosphate-activated protein kinase; ROS, reactive oxygen species; NF-κB, Nuclear factor kappa B.

Studies have demonstrated that several oncogenes, including *K-RAS* and *c-MYC*, induce intracellular ROS to promote cancer cell proliferation (67, 68). *K*-*RAS* also promotes extracellular ROS generation by increasing the activity of NOXs on the tumor cell membrane (69). In this regard, a study reports the tumor-promoting effect of autophagy in K-Ras [K-Ras(V12)]-induced malignant cell transformation, where inhibiting ROS with antioxidants reduced K-RasV12-induced induction of Atg5 protein and Atg7 protein, autophagy, and cancer growth (70). However, another study reports that rapamycin, an mTOR inhibitor, combined with (Hsp90) inhibitor IPI-504, causes tumor regression by promoting mitochondrial damage, oxidative stress, and autophagy in Kras/p53 mutant lung cancer and Nf1-deficient *RAS*-driven tumors (71).

Following another mechanism of action, ROS can also regulate autophagy through AMPK. AMPK induces autophagy during hypoxia or nutrient starvation by inhibiting mammalian target of rapamycin complex 1 (mTORC1 (72, 73). AMPK is phosphorylated by AMP-activated protein kinase kinase (AMPKK) following the accumulation of H_2_O_2_, which promotes its activation and autophagy induction (74). Inactivation of Atg4 protein increases autophagosomes and ATM-mediated oxidation of AMPK that inhibits mTORC1 in a H_2_O_2_-dependent manner (26, 75, 76). ROS can also mediate the induction of autophagy genes, including Beclin-1 or SQSTM1/p62, by regulating the activity of NF-κB in cancer cells (77–79).

The redox regulation of the proto-oncogene Akt provides another crucial point in the ROS-mediated regulation of autophagy. A well-described Akt-activating mechanism is PTEN oxidation (80, 81). In this regard, ROS production due to the growth factor stimulation promotes PTEN inactivation by forming a disulfide bridge between a cysteine in the catalytic site with a proximal cysteine residue. Consequently, Akt is activated due to increased PtdIns(3,4,5)P_3_ levels (81). However, disruption of mitochondrial membrane potential by an increase in H_2_O_2_ levels inhibits Akt, an upstream activator of mTOR, and induces autophagy (82; 83). This ROS-mediated signal transduction mechanism may also have a critical physiological role, as it may block catabolic pathways, like autophagy, in the presence of growth factors and may also induce the process of tumorigenesis.

Although ROS can promote autophagy induction, autophagy can also modulate ROS production. It was observed that caspase 8 inhibition and subsequent activation of JNK1 led to Atg6-Atg7 protein-dependent cell death when apoptosis was impaired (84). Moreover, caspase 8 inhibition promotes selective catalase degradation *via* autophagy that results in increased lipid peroxidation and autophagic cell death (85). Thus, it can be hypothesized that autophagy-mediated removal of catalase creates a self-sustaining loop, in which increased production of H_2_O_2_ by mitochondria may promote aberrant activation of autophagy, ultimately leading to autophagic cell death. However, catalase degradation was not observed under starvation conditions stimulating cytoprotective autophagy.

Furthermore, superoxides also modulate autophagy, as starvation-induced autophagy, mitochondrial electron transfer chain inhibitors, and the addition of exogenous H_2_O_2_ correlate with increased superoxide production and reduced H_2_O_2_ levels. Thus, overexpression of the SOD2 [manganese superoxide dismutase (Mn-SOD)] scavenges the superoxides, inhibits autophagy, and promotes H_2_O_2_ levels and starvation-induced cell death. In contrast, increasing superoxide levels by using the mitochondrial electron transfer chain inhibitors combined with SOD inhibitor 2-methoxyestradiol (2-ME) promoted both autophagy and cell death (86).

**Figure 2:**
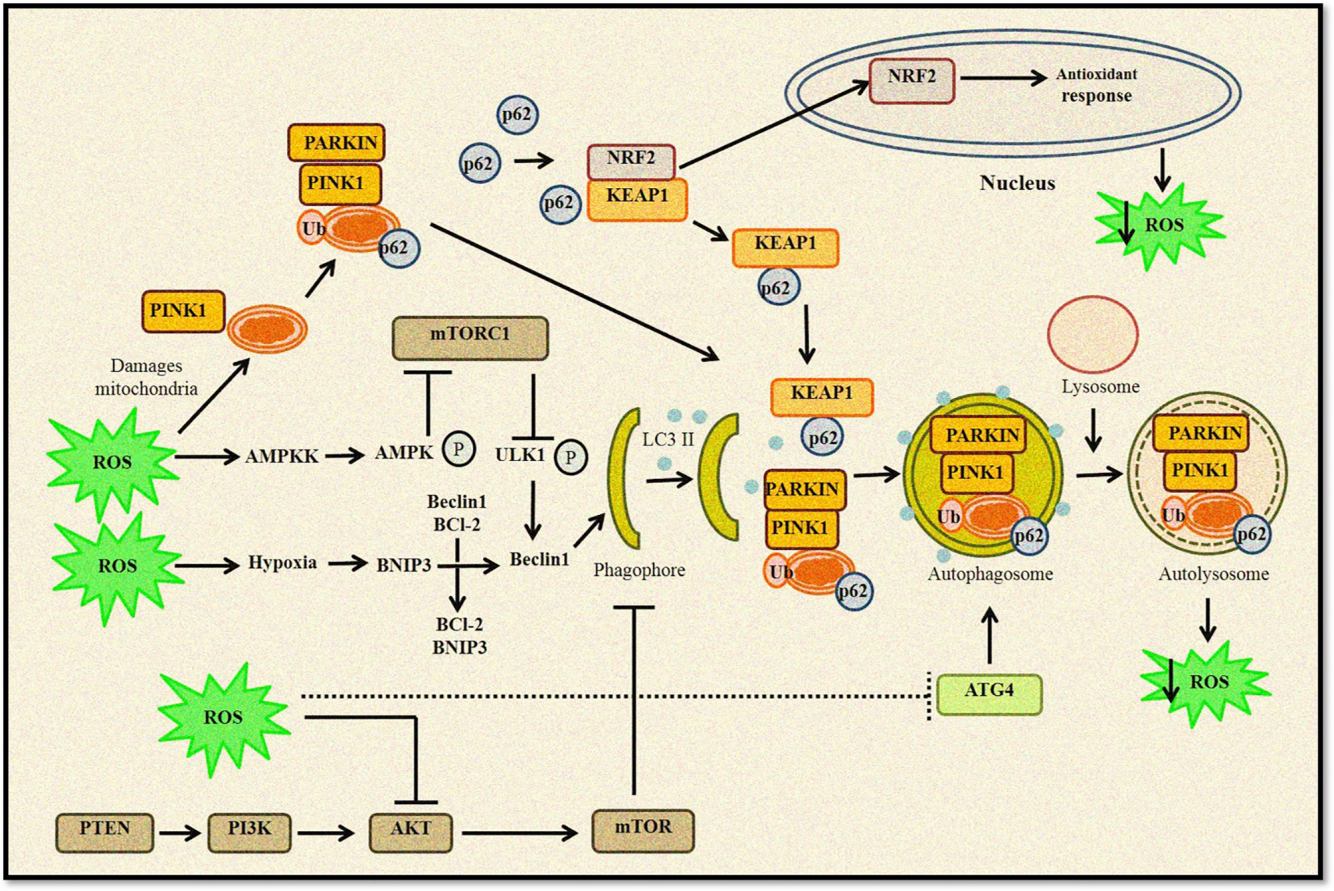
Relationship between ROS and autophagy. Increased ROS levels lead to oxidation of Atg4, which triggers autophagosome formation. ROS can regulate autophagy through AMPK activation that in turn phosphorylates ULK1 complex and promotes autophagy induction. Furthermore, disruption of Beclin 1–Bcl2 complex also induces autophagy. Any change in mitochondrial homeostasis promote ROS accumulation inducing mitophagy and removal of damaged mitochondria. Kelch-like ECH-associated protein 1 (KEAP1) degradation by p62-mediated selective autophagy leads to Nrf2-regulated antioxidant production and reduction in ROS levels. ROS can also inhibit the Akt/mTOR signaling cascade to induce autophagy.

Thus, it can be concluded that autophagy and ROS-generating agents work in an unprecedented complex manner, as ROS-induced autophagy and *vice versa* can either be a cytoprotective mechanism that reduces oxidative stress or a self-destructing process promoting autophagic cell death (**Figure 2**). A clearer understanding of this intricate crosstalk between autophagy and ROS can help develop therapeutic strategies and open several opportunities to target the disease development process.

## Autophagy and Reactive Oxygen Species in Cancer: A Promoter or Suppressor

Autophagy usually acts as a survival pathway in normal and cancer cells exposed to various stresses like hypoxia, nutrient deprivation, or chemotherapeutics. These stress conditions also promote ROS generation that could aid in autophagy-mediated cell survival (25, 86). Indeed, ROS accumulation can activate several transcription factors like p53, hypoxia-inducible factor-1 (HIF-1), nuclear factor (erythroid-derived 2)-like 2 (NRF2), and forkhead box transcription factors (FOXO3), which can increase the transcription of several proteins involved in autophagy (87). The initial connection between autophagy and cancer was established when studies demonstrated that Beclin-1 is mono-allelically deleted in approximately 50% of breast, ovarian, and prostate cancers (88, 89). Other studies revealed that mice hemizygous for Beclin-1 show a high incidence of lymphoma, liver, and lung cancer (90, 91).

Thus, it was believed that autophagy acts as a tumor suppressor. It removes damaged mitochondria through mitophagy and prevents ROS accumulation, therefore limiting the tumor-promoting effect of ROS (92). Consequently, autophagy inhibition promotes ROS production, mitochondrial impairment, and DNA damage, all potentially pro-tumorigenic during tumor initiation (6) but deleterious at later stages (75, 93). Studies have shown that autophagy loss causes genomic instability and aneuploidy (94, 95). Furthermore, autophagy dysfunction can promote tumor cell-extrinsic effects, including a pro-tumorigenic inflammatory microenvironment (25).

ROS are also induced by several tumor-associated immune cells in the tumor microenvironment (TME) (96) that may trigger altered activation of macrophages and immunosuppression (97). Macrophages are the first host cells to enter the TME to kill cancer cells (98). However, tumor-associated macrophages (TAMs) infiltrate into the tumors and differentiate into mature pro-tumor macrophages (M1 and M2 macrophages) mediated by cytokines in the TME (99–101). Although the pro-tumorigenic role of M1 is context dependent based on tumor microenvironmental cues (102, 103). Macrophages also show phagocytotic activity toward damaged tumor cells (104). However, macrophages are recruited through chemokines during cancer initiation, amplifying an inflammatory response. Macrophages also produce redoxosomes (exosomes containing functional NOX complexes) in the TME, which generates extracellular ROS and is incorporated into neighboring cells through endocytosis (105). Thus, a supportive TME is essential for tumorigenesis, wherein ROS plays a significant role in creating immunosuppressive TME for cancer growth and metastasis. Hence, it is plausible that autophagy inhibition may promote pro-tumorigenic ROS, since dysregulated autophagy leads to mitochondrial damage and high ROS levels, and oxidative stress, all potentially pro-tumorigenic.

Several studies have demonstrated that dysregulated autophagy due to the deletion of proteins such as Atg16L1, Beclin-1, or LC-3B promotes the accumulation of damaged mitochondria and mitochondrial ROS. It also promotes inflammation linked to increased levels of IL-1β and IL-18 (106–109). ROS can also be induced by IL-1, whose high expression has been associated with a poor cancer prognosis (110). Moreover, increased ROS levels also activate pro-inflammatory factors such as the pyrin domain-containing 3 (NLRP3) inflammasome (109). Inflammation aids in cancer initiation and survival through vascularization and stimulating the TME through the IL-1 and IL-18 pathway. Inflammatory cells further produce ROS or reactive nitrogen species (RNS) *via* iNOS, xanthine oxidase (XO), nicotinamide adenine dinucleotide phosphate (NADPH) oxidase, and myeloperoxidase (MPO). These oxidant-generating enzymes may promote damage to DNA damage. (111). Autophagy also plays a crucial role in inflammation by regulating the homeostasis, development, and survival of inflammatory cells (112). Inflammatory cells also release cytokines, activating NF-κB. NF-κB can help generate excess ROS or RNS by stimulating COX_2_, lipoxygenase (LOX), and inducible nitric oxide synthase (iNOS), that in turn may stimulate several oncogenes such as c-Jun and c-Fos and initiate tumorigenesis (113).

Another major regulator of both autophagy and ROS is the tumor suppressor p53 that plays a contrasting role in autophagy based on its subcellular localization (114). Nuclear p53 is suggested to activate autophagy through several transcriptional mechanisms. Many autophagy genes are said to be direct interacting partners of p53, and that autophagy helps in p53-dependent apoptosis and cancer suppression (115). In the nucleus, p53 activates the transcription of pro-autophagic molecules such as AMPK, damage-regulated autophagy modulator (DRAM), death-associated protein kinase 1 (DAPK-1), pro-apoptotic Bcl-2 proteins, sestrin 2, and Tuberous Sclerosis Complex 2 (TSC2) (116–120). However, cytoplasmic p53 inhibits autophagy, primarily through interactions with autophagic proteins (114). Cytoplasmic p53 mediates mitochondrial outer membrane permeabilization, promoting apoptosis and inhibiting autophagy (121, 122). Although the mechanism of cytoplasmic p53-mediated autophagy inhibition is not well elucidated, it was observed that cytoplasmic p53 inhibits AMPK and activates mTOR, leading to autophagy suppression (123).

p53 also can regulate autophagy by modulating ROS levels. During oxidative stress, basal p53 induces several antioxidants such as GPx1, MnSOD, ALDH4, and TPP53INP1 to remove oxidative stress (124–127). Additionally, p53 also exerts antioxidant effects by upregulating the expression of several p53 target genes in response to DNA damage and oxidative stress. This leads to inhibition of mTORC1 activity and autophagy induction. Sestrin1 and sestrin2 are the links between p53 activation and mTORC1 activity (119). Sestrins also regulate ROS (128) and inhibit mTORC1 activity by inducing the expression of the pro-autophagic AMPK, TSC1, and TSC2 (119).

However, p53 can also induce ROS. A study observed that silibinin, an active constituent extracted from *Silybum marianum* (milk thistle), induced ROS-mediated autophagy and apoptosis in HeLa cells (129). Furthermore, another study by the same group demonstrated that silibinin promotes p53-mediated ROS in HeLa cells. The study also observed that p53 inhibition decreased ROS generation and reversed silibinin’s growth-inhibitory effect. Moreover, silibinin was not able to induce ROS in the epithelial carcinoma cells (A431), as they lack p53 activity (p53His273mutation) (130). Another study reports that silibinin may upregulate p53-mediated autophagy by inhibiting MAPK and PI3K/Akt pathways and activating ROS/p38 and JNK pathways (131). Furthermore, upregulation in PI3K and AKT or downregulation in PTEN activates mTOR and inhibits autophagy. Thus, these oncogenic alterations suggest the importance of autophagy suppression during tumor initiation (132, 133).

Other studies also demonstrated that any defect in the autophagic machinery promotes tumor initiation, including liver and breast (114, 134). Tang etal. (114) demonstrated that low expression of Beclin-1 suggested poor prognosis in Her2, basal-like, and p53-mutant breast cancer. Autophagy also acts as a tumor suppressor through its role in cellular senescence, where cells undergo growth arrest (135). Kang etal. (136) demonstrated that GATA Binding Protein 4 (GATA4), a transcription factor regulating senescence, is degraded by p62-selective autophagy. Autophagic adapters, p62/SQSTM1, act as cargo receptors for autophagic degradation of ubiquitinated targets (137). p62 is upregulated under various stresses, including ROS, where ROS-induced p62 gene expression is mediated by NRF2. Furthermore, p62 protein activates NRF2 by interacting with the Nrf2-binding site on Keap1, a component of Cullin 3 (CUL3)-based E3 ubiquitin ligase for Nrf2, resulting in stabilization of Nrf2 and transcriptional activation of its target genes (138, 139). Another major autophagy regulator, Atg5 protein, also plays a dual role in the regulation of autophagy and apoptosis. Studies have indicated that overexpression of Atg5 protein can sensitize tumor cells to chemotherapy. In contrast, silencing the *ATG5* gene with short interfering RNA made tumor cells partially resistant to chemotherapy. Atg5 protein is cleaved by calpains, a family of Ca2+-dependent cysteine proteases, producing an amino-terminal cleavage product. Calpain induction and subsequent Atg5 protein cleavage appear to be universal phenomena in apoptotic cells (140). Similarly, the Atg12 protein also has a dual function, participating in both autophagy and apoptosis, and is necessary for caspase activation in response to a range of apoptotic stress inducers. Non-conjugated Atg12 protein can bind to and inhibit Mcl-1 and Bcl-2 by a BH3-like motif, inducing mitochondria-dependent apoptosis (141). Knockout of *ATG12* gene prevents Bax activation and cytochrome c in apoptotic cells.

Although autophagy functions as a tumor suppressor during the initiation of tumorigenesis (6), other studies have revealed that autophagy can also act as a tumor promoter (132; 142). Furthermore, autophagy can also promote resistance to many anticancer therapies (27). The pro-survival role of autophagy can be seen during stress conditions, including hypoxia and nutrient deprivation. Autophagy rapidly degrades unfolded proteins during stress and provides the substrate for ATP production (143, 144). Thus, autophagy is generally upregulated in hypoxic regions of a tumor and promotes cell survival (25).

During later stages of tumor initiation, autophagy is required for cell transformation by the RAS oncogene to promote cell tolerance to stress A high basal level of autophagy is observed in RAS-mutated cancers, including lung, colon, and pancreatic (145, 146). Furthermore, mutations in the RAS genes promote uncontrollable cell proliferation and apoptosis inhibition (147, 148). Herein, autophagy promotes cancer cell survival by providing nutrients during starvation or other stress conditions (149). Consequently, autophagy inhibition increases the accumulation of damaged mitochondria and promotes cell death (150). Thus, tumor cells utilize autophagy to survive metabolic stress, and autophagy mitigates cellular damage (151). Autophagy inhibition leads to slower tumor growth and increased sensitivity to cancer treatments. This has led researchers to assess the efficacy of autophagy inhibitors combined with chemotherapy to increase therapeutic responses in cancers.

Consistently, autophagy inhibition reduced malignant transformation and proliferation of mouse embryonic fibroblasts (MEFs) transformed with Harvey Rat Sarcoma Virus (HRAS) and MDA-MB-231 breast cancer cells presenting with KRAS expression (152). Other studies have shown that model systems such as immortalized baby mouse kidney (iBMK), MCF-10A, and pancreatic ductal adenocarcinoma (PDAC) cell lines harboring ectopic expression of the oncogenic KRAS has high basal autophagy levels. However, inhibiting autophagy by deleting the gene *ATG5* or *ATG7* prevented RAS-mediated cancer cell proliferation (145; 70, 153). It can be stated that mitochondrial respiration is required for RAS-induced tumorigenesis, and active autophagy maintains cellular homeostasis (154). Thus, RAS-mediated cancers are addicted to autophagy for survival, and dysregulated autophagy in these cancer types is proportional to decreased cancer cell survival, accumulation of damaged mitochondria, and oxidative stress that may ultimately promote cell death (155; 25). Furthermore, p62/SQSTM1 deficiency also reduces tumorigenicity and increases ROS levels following RAS activation (145, 156, 157). Another study also states that autophagy inhibition by FIP200 (FAK family-interacting protein of 200 kDa) deletion suppressed the breast cancer initiation *in vivo* driven by the polyoma virus middle T (*PyMT*) oncogene. The study demonstrated that FIP200 ablation promoted accumulation of p62/SQSTM1, ubiquitinated protein aggregates, and deficient LC3 conversion with an increased number of abnormal mitochondria confirming the pro-tumorigenic role of autophagy (158). Interestingly, FIP200 deletion did not affect apoptosis but significantly reduced the proliferation of breast cancer cells or Ras-transformed MEFs.

**Figure 3:**
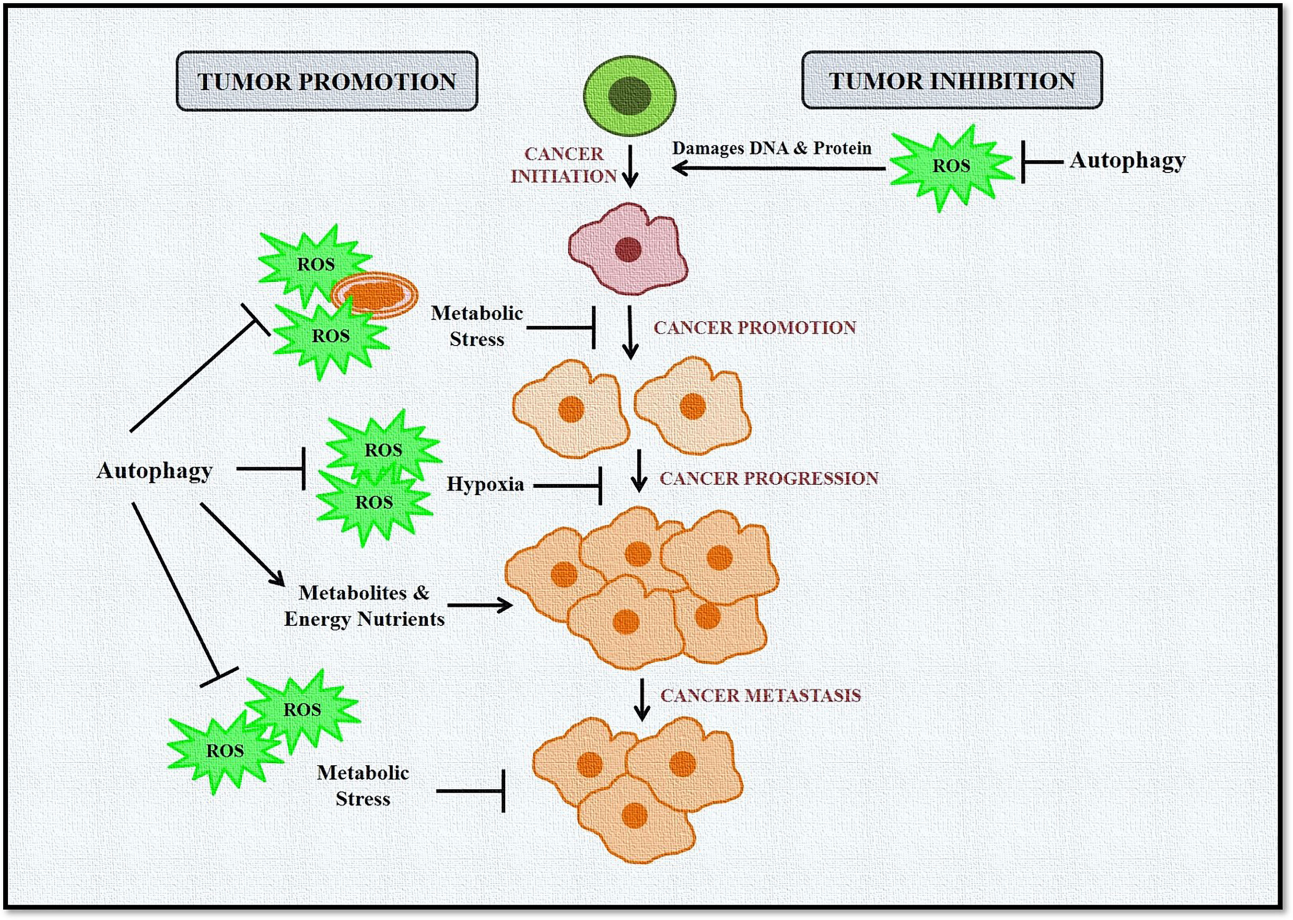
Role of autophagy and ROS in cancer promotion and suppression. Autophagy in cancer works in a context-dependent manner based on tumor type and stage. It acts as a suppressor during tumor initiation but plays a protective role in established tumors. During tumor initiation, autophagy targets ROS-damaged organelles, DNA, and protein toward degradation, leading to inhibition of tumorigenesis. Autophagy eliminates ROS-induced stress during tumor progression and metastasis and provides much-needed nutrients to cells, including cancer cells. ROS is also induced in cancer cells during hypoxic conditions, activating autophagy in stromal cells. These cells then provide high-energy nutrients for cancer cell survival.

Taken together, these studies confirm the complex and paradoxical role of autophagy and ROS in cancer initiation and progression (**Figure 3**). However, this dual role also provides several therapeutic windows that could be exploited to develop targeted anticancer therapies.

## Role of Autophagy and Reactive Oxygen Species in Epithelial to Mesenchymal Transition and Cancer Metastasis

Metastasis is a complex mechanism in which cancer cells undergo epithelial to mesenchymal transition (EMT) and spread from the tissue of origin to distant organs. It is the main reason behind high cancer mortality (159–161). EMT promotes contact inhibition in cancer cells, leading to invasive tumor epithelial phenotype (162). EMT can be regulated by several mechanisms, including epigenetics, transcriptional control, miRNAs, protein stability, alternative splicing, ROS, and autophagy (163, 164).

A study by Avivar-Valderas etal. (165) observed that in mammary tumor cells, autophagy was induced due to matrix detachment or integrin blockade in response to ROS-dependent upregulation of protein kinase R-like ER kinase (PERK1). Consistently, autophagy or PERK inhibition during matrix detachment or integrin signaling blockade induced cell death and reduced clonogenic recovery following detachment, highlighting the role of PERK-induced autophagy in mammary tumor cell survival during matrix detachment (165, 166). Furthermore, hepatocellular carcinoma and melanoma cells also require autophagy to survive following matrix detachment, leading to increased lung colonization during metastasis (167–169). Moreover, high ROS levels induced by matrix detachment may further promote autophagy activation through direct activation of Atg4 protein (26, 170).

One of the major contributors of EMT is transforming growth factor-beta 1 (TGF-β1) (171). Exogenous TGF-β1 regulates urokinase-type plasminogen activator (uPA) and Matrix metalloproteinase 9 (MMP9) to promote cell migration and invasion by activating NF-κB *via* the Rac1-NOXs-ROS-dependent mec`ism (172). ROS also regulates EMT *via* the non-canonical TGF-β1–TGF-β-activated kinase 1 (TAK1) pathway. TAK1 deficiency promotes integrin:Rac-induced ROS, further accelerating the EMT process. Consistently, low TAK1 expression was observed in invasive squamous cell carcinoma (SCC) but not in benign SCCs (173). ROS-mediated activation of Nrf2 also promotes Notch signaling and EMT induction (174). ROS can also activate TGF-β1 in response to ionizing radiation (175). Thus, these studies significantly highlight the role of ROS in EMT induction. Moreover, it is well characterized that cancer cells have a high metabolic rate. Therefore, to fulfill the bioenergetic needs of the cancer cells, an increase in ATP production and tricarboxylic acid (TCA) cycle is required. In turn, ROS is accumulated due to increased oxidative metabolism, disturbing the cellular homeostasis, dysregulating autophagy, inducing EMT, and promoting cancer cell survival and metastasis (6, 176, 177).

Furthermore, self-aggregation of TGF-β1-induced antiapoptotic factor (TIAF1) was observed in the cancer stroma and peritumor capsules of solid tumors, which is indicative of aggregation-dependent control of cancer progression and metastasis (178).

Autophagy also helps tumor cells adapt to hypoxic conditions before vascularization during *in vivo* tumor formation (179). High autophagy levels were observed in the hypoxic regions of the tumors. Autophagy can also be activated by ischemia to promote cancer cell survival and growth (25, 94, 95). Moreover, hypoxia can also induce ROS and stabilizes HIF-1α, the primary regulator of oxygen homeostasis (180). HIF-1α induces mitophagy *via* Bcl-2/adenovirus E1B 19-kDa-interacting protein 3 (BNIP3), along with a constitutive expression of Beclin-1 and Atg5 protein promotes cell survival during prolonged hypoxia by preventing increased ROS levels (181). BNIP3, a target gene for HIF-1α, induces autophagy by disrupting the Beclin 1–Bcl2 interaction (182). Autophagy dysregulation due to *BECLIN-1*, *ATG5* gene, or *ATG7* gene knockdown promotes hypoxia-induced cell death. Indeed, BNIP3-induced autophagy is required to prevent aberrant ROS levels during hypoxia and thus presents a survival mechanism (183–185). Autophagy is also induced in a HIF-1α-independent manner *via* AMPK and unfolded protein response (UPR) during hypoxia (186, 187).

Starvation-induced autophagy can also induce EMT and is required for HepG2 and BEL7402 HCC cell invasion *in vitro*. Thus, knockdown of autophagy genes like *ATG7* or *ATG3* in these cells suppressed EMT and invasion and decreased the expression of Fibronectin 1 (FN1), TGF-β1, and activated SMAD family member 3 (SMAD3) (188). Kim etal. (189) observed that another autophagy regulator, Unc-51 Like Autophagy Activating Kinase 2 (ULK2), promotes EMT by downregulating E-cadherin and increasing the invasiveness of lung cancer cells *in vitro*. Increased autophagy also promotes mesenchymal stem-like phenotype and invasion/migration of glioblastoma stem cell lines. Hence, autophagy dysregulation *via ATG12* gene knockdown or p62/SQSTM1 deficiency reduced invasion and migration phenotypes in glioblastoma cells (190, 191).

Contrarily, another study argues that autophagy reduces migration of glioblastoma tumor cells *via* SNAIL and SLUG inhibition (192). Similarly, in hepatocytes, autophagy inhibition *via* liver-specific knockout of *ATG7* gene (*Alb-Cre*;*Atg7^fl/fl^*) promoted the expression of vimentin and SNAIL. The study further reports that autophagy degraded Snail in a p62/SQSTM1-dependent manner. Moreover, treating wild-type MMH (murine hepatocytes) with TGF-β1 suppressed autophagy, whereas starvation-induced autophagy inhibited TGF-β1-mediated EMT (193).

Low basal autophagy levels also correlate with an increased propensity for migration and invasion in Skov-3 ovarian cancer cells compared to cells with high basal autophagy. Furthermore, a decrease in migration, invasion, and expression of the mesenchymal markers was observed due to starvation-induced autophagy, which was reversed following siRNA-mediated knockdown of *ATG7* gene. Moreover, EMT transition in these cells was regulated *via* increased ROS and heme oxygenase 1 (HMOX1), highlighting a role of autophagy in the ROS–HMOX1–EMT signaling axis (194). Similarly, autophagy can also inhibit EMT by degrading SNAIL and TWIST, two major mesenchymal markers that promote the invasion phenotype in cancer cells (195). Apart from TGF-β1, EMT is also induced by IL-1, IL-6 that regulate SNAIL or TWIST. ROS also induces HIF-1α and lysyl oxidase (LOX), decreasing E-Cadherin levels and activating EMT and cancer cell migration. Thus, it is plausible that autophagy may also be detrimental to EMT by inhibiting inflammation and removing ROS (196).

The autophagy receptor p62/SQSTM1 stabilizes the transcription factor TWIST and induces EMT (197, 198). Autophagy inhibition also promotes p62/SQSTM1 accumulation and contributes to tumorigenesis. Autophagy loss promoted the expression of TWIST in a p62-dependent manner, where it directly binds to TWIST and prevents its proteasomal degradation, promoting EMT and metastasis *in vivo* (197). Another study also demonstrated that accumulation of p62/SQSTM1 stabilizes TWIST and activates TGF-β1–SMAD signaling, further promoting EMT-associated junction remodeling (198).

**Figure 4:**
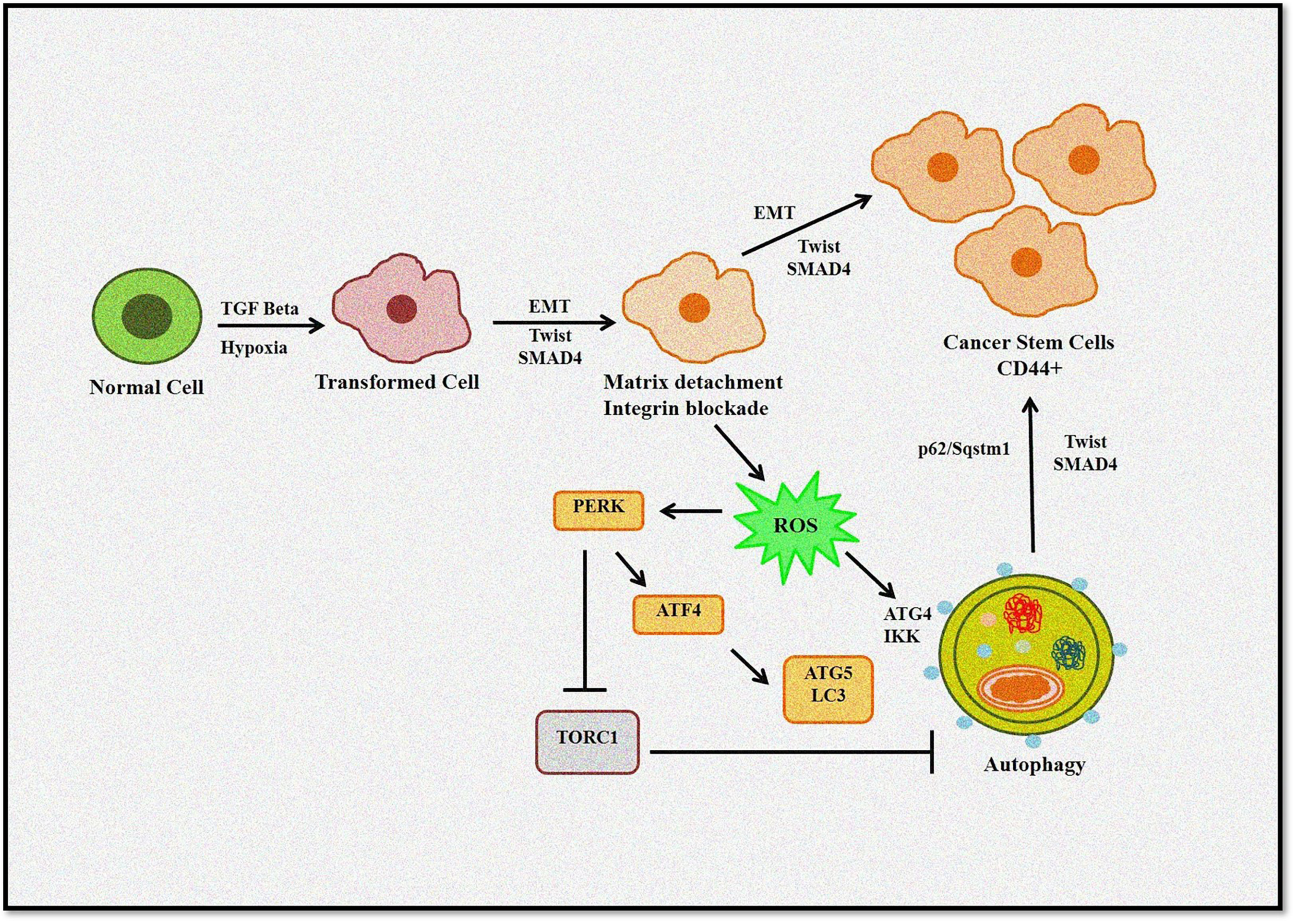
Role of autophagy and ROS in the EMT process. Autophagy induces tumor invasiveness by promoting stem cell phenotype linked to hypoxia and TGF-β. Matrix detachment leads to ROS-induced EMT transition and autophagy induction. Furthermore, p62/Sqstm1 autophagy cargo adapter interacts with Twist, an EMT regulator, preventing its proteasomal degradation and promoting invasion.

It is evident that a complex link exists between autophagy, ROS, and EMT (**Figure 4**). Thus, to design better treatment modalities, extensive knowledge of the interlinked cellular events would be necessary to regulate cellular homeostasis.

## Role of Autophagy and Reactive Oxygen Species in Cancer Therapy

For the past two decades, autophagy has been an attractive target for researchers to develop better anticancer therapies. Several cancer drugs either induce cytoprotective autophagy or promote autophagic cell death or autophagy-mediated apoptosis in cancer cells. Indeed, the cytoprotective role of autophagy was observed against temozolomide (199), tamoxifen (200), the histone deacetylase inhibitor SAHA (201), cyclophosphamide (27), irradiation (202), imatinib mesylate (203), and cisplatin (204). Thus, autophagy inhibitors such as hydroxychloroquine were used combined with standard chemotherapeutics in clinical trials to increase the therapeutic potential of the drugs (205). However, it should be noted that the stage at which autophagy is inhibited may alter drug sensitivity and plays a critical role in deciding the fate of cancer cells.

Certain anticancer treatments also promote ROS-induced autophagy that can promote drug resistance. In this case, using autophagy inhibitors with the chemotherapy agents may help restore the sensitivity to the treatment. Moreover, the type and dosage of drugs used, along with the cancer genotype, are other factors that may decide the outcome of autophagy activation. Consistently, Beclin-1-dependent protective autophagy was induced when pancreatic cancer cells were exposed to sorafenib, a pan-kinase inhibitor combined with HDACI, a histone deacetylase inhibitor. However, Bcl-2 knockdown or inhibition conditioned Beclin 1-dependent autophagy to promote apoptosis into a toxic pathway promoting intrinsic apoptosis (206). Another study demonstrated that ROS-mediated activation of c-Jun N-terminal kinase (JNK) induced cytoprotective autophagy when human rhabdomyosarcoma (Rh30 and RD) cells were treated with ciclopirox olamine (CPX). However, inhibiting autophagy *via* chloroquine (CQ) promoted CPX-induced cell death (207).

Hahm et al. (208) reported that honokiol, derived from the bark of *Magnolia officinalis*, induced ROS-induced cytoprotective autophagy and promoted drug resistance in prostate cancer. However, inhibiting autophagy *via* 3-methyladenine (3-MA) or *ATG5* gene siRNA sensitized cancer cells to apoptosis (208). Moreover, exposing breast and glioblastoma cancer cells to mitoquinone and quercetin, respectively, also promoted cytoprotective autophagy (209, 210). Hence, it can be hypothesized that any changes in the mitochondrial homeostasis would induce ROS and autophagy, which may lead to cell survival by autophagy-mediated degradation of damaged mitochondria. Therefore, autophagy inhibitors or siRNA-mediated silencing of *ATG* genes can turn protective ROS deleterious to cancer cells and promote apoptosis.

Another study showed that using 3-bromopyruvate (3-BrPA), a hexokinase II inhibitor, induced autophagy in breast cancer cells (MDA-MB-231435 and MDA-MB-435). However, ROS-mediated cell death was observed when 3-BrPA was used in combination with chloroquine, an autophagy inhibitor. The authors also concluded that autophagy induction was not dependent on ROS accumulation (211). Similar results were observed when A549 lung cancer cells were exposed to artemisinin, an antimalarial drug. Treatment with artemisinin induced autophagy that was attenuated by chloroquine. Autophagy inhibition promoted the accumulation of damaged mitochondria and ROS generation, resulting in apoptosis. Furthermore, apoptosis was ROS-dependent, as using a ROS scavenger N-acetyl-cysteine (NAC) rescued A549 cells from apoptosis *via* caspase-3 inhibition (212).

However, autophagy-induced apoptosis has also been reported. Carnosol, a polyphenol, inhibited the cell viability in MDA-MB-231 breast cancer cells. The study reported that carnosol caused DNA and mitochondrial damage and promoted ROS-dependent early autophagy and late apoptosis (213). Thus, this could be another mechanism of action that a drug could follow to induce cancer cell death. Some chemotherapy agents like 2-methoxyestradiol (2-ME) and arsenic trioxide (As_2_O_3_) also promote oxidative stress-mediated autophagic cell death (214). Nevertheless, ROS is essential for As_2_O_3_-mediated autophagic cell death in glioma cells (215). 2-ME also upregulates ROS levels by inhibiting complex I of the mitochondrial electron transport chain and mitochondrial SOD (77, 216, 217). Furthermore, 2-ME, a ROS-generating agent, induced autophagic cell death in a transformed cell line HEK293 and the cancer cell lines HeLa and U87 (77). However, both 2-ME and As_2_O_3_ can induce autophagy and apoptosis (17, 215).

Autophagy-induced apoptosis was also observed in A375, HT144, and Hs294T cells treated with the H1 histamine receptor antagonist terfenadine, which may increase ROS depending on culture condition (218). Similarly, in melanoma cancer cells (A375 and BLM), bortezomib, a proteasome inhibitor, at least in part *via* ROS-mitochondrial dysregulation-associated pathways (219). Another study revealed that sasanquasaponin III (SQS III) inhibited the viability of A375 cells by inducing apoptosis and autophagy. The authors further observed that both, apoptosis as well as autophagy induction was ROS dependent. (220). Moreover, resveratrol and psoralidin promoted ROS-triggered autophagy induction followed by apoptosis in colon and lung cancer cell death, respectively (221, 222).

Other studies also highlight the role of autophagy and ROS levels in cancer treatment. It was shown that 2-deoxy-D-glucose (2DG), when combined with cisplatin or staurosporine, promoted apoptosis but promoted cytoprotective autophagy and decreased ROS levels when combined with pyrimethamine. Moreover, 2DG alone promoted protective autophagy, inhibited ROS levels, and increased mitochondrial membrane potential in melanoma cells (8863 and 501) (223, 224).

Thus, several treatment studies can be used to induce cancer cell death. As cancer develops high resistance against apoptosis, causing autophagic cell death could be an option. Moreover, combining ROS and autophagy-inducing agents could also promote cancer cell death. Other strategies include combining apoptosis inducers with autophagy inhibitors in cancer cells harboring protective autophagy (**Table 2**). Taken together, choosing correct cancer treatment strategies is highly complex and should be based on tumor phenotype and genotype.

**Table 2:** ROS-inducing or -inhibiting chemotherapeutic agents and their effect on autophagy.

	Drug	Cancer type	Mechanism of action	Reference
1	Arsenic trioxide	Ovarian cancer cells (HEY, OVCA429, and SKOV3)	Induced Beclin 1-independent autophagic pathway by modulating SnoN/SkiL expression and altering TGFβ signaling *via* ROS generation	(225)
2	Artemisinin	Different cancer cells	Weakens the levels of glutathione, elevates ROS levels, and Self-amplification of oxidative stress Induces cytoprotective autophagy	(212, 226)
3	Buthionine sulfoximine	Phase I/II studies	Inhibitor of GSH synthesis	(227, 228)
		Cancer cells (Human gallbladder cancer (GBC-SD), human cholangiocarcinoma (RBE) and osteosarcoma cells (DLM8 and K7M3)	Depletes intracellular GSH; increased apoptosis may affect the STAT3 pathway, induces oxidative stress and autophagy	(226, 229, 230)
4	b-Lapachone (ARQ501)	Pancreatic cancers, squamous cell carcinoma and glioma cells	Produces ROS by undergoing futile redox-cycles catalyzed by NQO1 Induces autophagic cell death in glioma cells	(231–233)
5	Chloroquine	Cancer cells (MCF-7, HT29, U373)	Inhibition of autophagy; increased ROS generation and subsequent cell death	(183)
6	Cisplatin	Head and neck cancer patients Bladder cancer cells	Induced ROS levels and DNA damage Induces cytoprotective autophagy	(234, 235)
7	Curcumin	Colon cancer cells (HCT116)	Induced ROS production and autophagic cell death	(236)
8	Daunorubicin	T-lymphoblastic leukemia cells (CCRF-CEM and MOLT-4), B-lymphoblastic leukemia cells (SUP-B15) and Chronic myelogenous leukemia (K562 cells)	Increased expression of SOD2 and lower ROS production Induces cytoprotective autophagy	(237, 238)
9	Doxorubicin (Adriamycin)	Different cancers	Cell death through multiple intracellular targets: ROS generation, DNA adduct formation, topoisomerase II inhibition, histone eviction, Ca^2+,^ and iron hemostasis regulation, and ceramide overproduction. Inhibits autophagy to induce cancer cell death	(239, 240)
10	Diphenylene iodonium	Pancreatic cancer Colon cancer cells (HT-29), colon cancer cells (HCT-116) Macrophages	Jak/STAT pathway inhibited dephosphorylation of AKT/ASK1 pathway and low ROS levels promotes apoptosis Inhibit ROS level Inhibits autophagy in macrophages	(241–243)
11	Disulfiram	Advanced non-small lung cancer carcinoma, Metastatic melanoma cells (c81-46A, c81-61, and c83-2C) Lung cancer Pancreatic, breast and colorectal cancer cells	Inhibitor of cytosolic SOD1 Induces cytoprotective autophagy in lung cancer Induces autophagy-dependent apoptosis in pancreatic and breast cancer cells Induces autophagic cell death in colorectal cancer cells	(244–248)
12	Fullerene C60 (Nano-C60)	Normal and drug-resistant cancer cells MCF-7 and HeLa)	Induced autophagy and sensitizes chemotherapeutic agents to kill drug-resistant cancer cells in a ROS-dependent and photo-enhanced fashion	(249)
13	Gemcitabine	Head and neck cancer, pancreatic cancer Triple-negative breast cancer cells (TNBC), bladder cancer	Activate antioxidant agents, suppress Nox4, block ROS-related signaling pathways Induces cytoprotective autophagy in TNBC, pancreatic cancer, and bladder cancer	(234, 250–253)
14	Idarubicin (IDR)	Breast cancer, cardiac muscle cell (HL-1) Leukemia (K562 cells)	Induces ROS, oxidative DNA damage, and apoptosis Induces autophagy and promotes apoptosis in leukemia	(254–256)
15	Imexon	Phase I/II studies leukemia	Binds to thiol to disrupt GSH activity elevate oxidative stress and stimulate apoptosis in cancer cells.	(257, 258)
16	Itraconazole	Liver cancer, glioblastoma, colon cancer	Increases ROS and activates apoptosis in liver cancer Induces autophagic cell death in glioblastoma Induces autophagy-mediated apoptosis in colon cancer	(259–261)
17	Mangafodipir	Cancer cell line (CT26, Hepa1.6, and A549)/Phase II studies in combination with chemotherapy in liver cancer	Increased H_2_O_2_ levels, specifically in cancer cells. SOD, catalase, and GSH reductase mimetic	(262)
18	Medroxyprogesterone	Head and neck cancer Glioblastoma	Induction of 15d-PGJ2-ligand of PPAR, increased ROS and Induced apoptosis Induces autophagy in C6 glioma cells when used in combination with tibolone or temozolomide	(234, 263, 264)
19	Metformin	Colorectal, Pancreatic cancer, Hepatocellular carcinoma, preneoplastic JB6 Cl 41-5a cells	Increases ROS production Induces autophagy to promote cell death in pancreatic, hepatocellular carcinoma and preneoplastic cells	(265–268)
20	Motexafin gadolinium (gadolinium texaphyrin)	Hematological malignancies	Inducer of superoxide by futile redox cycling, an inhibitor of Trx, induces apoptosis in lymphoma cells.	(269; 144)
21	OSU-03012 (celecoxib derivative)	Hepatocellular carcinoma	Caused ROS accumulation and subsequent autophagic cell death	(270)
22	Panitumumab (EGFR antibody)	EGFR-expressing metastatic colorectal carcinoma	ROS accumulation and autophagic cell Death	(271)
23	Proton pump inhibitor esomeprazole	Melanoma	Induced ROS and protective autophagy	(272)
24	Photodynamic therapy (PDT)	Head and neck, brain, lung, bile duct, esophagus, bladder, ovarian, skin, ophthalmic, pancreatic, cervical, colorectal, and bladder carcinoma	Photochemical generation of cytotoxic ROS through the light-activation of a photosensitizer accumulated in cancer cells or tumor vasculature Induces cytoprotective autophagy	(273–277)
25	Proscillaridin A (PSD-A)	Breast cancer, colorectal cancer	ROS generation, Ca^2+^ Oscillation, inhibits STAT3 activation, induces apoptosis and Autophagy	(278)
26	Recombinant human HMGB1	Glioblastoma Pancreatic cancer	Activate MAPK and NF-κB, release cytokines, and induce NADPH oxidase to produce ROS. Induces cytoprotective autophagy in pancreatic cancer	(279–281)
27	Resveratrol	Colon cancer cells	Induced ROS and subsequent cytotoxic autophagy	(222)
28	Ruthenium(II) complexes	Cancer cells	Induced ROS and subsequent protective autophagy along with apoptosis	(282)
29	Suberoylanilide hydroxamic acid (Zolinza, Vorinostat)	Cutaneous T-cell lymphoma	Induced ROS and autophagy, prosurvival	(283, 284)
30	Sulforaphane	Therapy-resistant pancreatic carcinoma cells	Promoted mitochondria-derived ROS to initiate diverse cellular responses, including protective autophagy	(285, 286)
31	Sulindac	colon and lung cancer	mitochondrial damage, elevate ROS production and induces cytoprotective autophagy	(287, 288)
32	Tamoxifen	Breast cancer cells (MCF-7)	Induced ROS and subsequent protective autophagy	(289)
33	Temozolomide	Human glioblastoma cell lines (U87 MG, GBM8401, and GBM-SKH)	Induced ROS/ERK-mediated autophagy, protected glioma cells from apoptosis	(290)
34	Tetrathiomolybdate (ATN-224)	Phase II studies in myeloma, melanoma, prostate, and breast carcinoma	Inhibitor of cytosolic SOD1 copper chelation *via* tetrathiomolybdate induces cytoprotective autophagy in pancreatic cancer cells	(291–293)
35	Valproic acid	Glioma cells	Oxidative stress activated the ERK1/2 pathway, autophagic cell death	(294)
36	Vitamin A	Testis tumor Leydig cell lines	Modulated antioxidant enzyme activities, induced protective autophagy or apoptosis at different doses	(295)
38	2-Methoxyestradiol	Phase II studies in different tumors, Chondrosarcoma	Generates superoxide by inhibition of SOD Induces autophagy in chondrosarcoma whose inhibition promotes apoptosis	(296, 297)
39	7-formyl-10- methylisoellipticine	Acute myeloid leukemia	Increase mitochondrial ROS production and apoptosis induction	(298)

## Conclusion

Thus, it can be concluded that ROS and autophagy work in a tight regulation with each other to maintain cellular homeostasis. They can either help cancer cells adapt to severe stress, which may otherwise be detrimental to cells, or induce cell death. This paradoxical role of ROS and autophagy in cancer is mainly dependent on the cancer types and their microenvironment. Therefore, it is imperative to decipher the crosslinked mechanisms in tumorigenesis with respect to ROS and autophagy so that autophagy modulators may be designed to target cancer.

This review highlights the role of ROS and autophagy in cancer survival and suppression mechanisms. The major mechanisms include response to hypoxia, turnover of antioxidant enzymes, oxidative damage-induced protein aggregation of regulatory molecules like TGF-β1, p53, enhanced survival in RAS-mutated cancers, EMT transition, and drug resistance. However, consistent with the role of autophagy and ROS in cancer, they provide large windows of opportunities to develop better treatment strategies that may help fulfill the unmet needs of cancer patients.

A better understanding of the molecular and chemical mechanisms of the redox regulation of autophagy is required. There are still some unanswered questions like 1) How does autophagy modulate the turnover of regulatory enzymes required for maintaining redox potential? 2) How do autophagy and ROS regulate the posttranslational modifications of specific tumor suppressors? 3) How does excessive ROS impair autophagy and dysregulate the cellular microenvironment to promote invasive phenotype? Answer to these questions may help develop better anticancer treatment options.

## Author Contributions

AH: conceptualization, writing—original draft, writing—review and editing. SFR: preparation of figures. SP: preparation of tables. NP: critical revision of the article. AN: critical revision of the article. SSM: conceptualization, supervision, writing—original draft, writing—review and editing, critical revision of the article. All authors contributed to the article and approved the submitted version.

## Conflict of Interest

The authors declare that the research was conducted in the absence of any commercial or financial relationships that could be construed as a potential conflict of interest.

## Publisher’s Note

All claims expressed in this article are solely those of the authors and do not necessarily represent those of their affiliated organizations, or those of the publisher, the editors and the reviewers. Any product that may be evaluated in this article, or claim that may be made by its manufacturer, is not guaranteed or endorsed by the publisher.
